# Novel *WT1* and *ACTN4* co-mutations in a patient with Denys-Drash syndrome and an atypical, potentially attenuated presentation of nephropathy: a case report

**DOI:** 10.1186/s12882-025-04433-4

**Published:** 2025-09-01

**Authors:** Eric Frazier, Ryan Sabour, Matthew D. Nguyen, Dao Le, Ramy Hanna

**Affiliations:** 1 University of California - Irvine - School of Medicine, Irvine, CA USA; 2 Division of Nephrology, Hypertension, and Kidney Transplantation, Department of Medicine, University of California, Irvine, 3800 Chapman Ave, Suite 6200, Orange, CA 92868-3298 USA

**Keywords:** ACTN4, Wilms tumor, Denys–Drash syndrome, Nephropathy, Proteinuria, Case report

## Abstract

**Background:**

Denys-Drash syndrome (DDS) is defined by early onset nephrotic syndrome rapidly progressing to end stage renal disease (ESRD) before 4 years of age, male pseudohermaphroditism, and Wilms tumor (WT). DDS is associated with mutations in the *WT1* gene, most commonly in exons 8 or 9. *ACTN4* mutations are associated with nephrotic syndrome and renal dysfunction, with an onset in early adulthood.

**Case presentation:**

We present the case of an 18-year-old male with a past medical history of Wilms tumor (status post right nephrectomy, chemotherapy, and radiation during infancy), Denys-Drash syndrome, chronic kidney disease stage 2, and autism spectrum disorder. The patient presented to our clinic with worsening proteinuria discovered secondary to pyelonephritis. Genetic evaluation revealed a *WT1* mutation, c.388_389insAC (p.Pro130Hisfs*34), complicated by an *ACTN4* mutation, c.2698T > A (p.Ser900Thr). Under our care, his worsening proteinuria stabilized, and his estimated glomerular filtration rate (eGFR) remained at 83 mL/min/1.73 m^2^ (1.38 mL/s/1.73 m^2^), indicating preserved renal function. We used a multidisciplinary approach to manage this patient through lifestyle modification, regular monitoring, and conservative measures. Surveillance with regular ultrasounds and labs has been key in management. We chose to forgo biopsy because of the risk to the remaining kidney, and we will continue to evaluate the need for an angiotensin-converting enzyme (ACE) inhibitor on the basis of hemodynamic stability.

**Conclusion:**

In this case, we present a patient with delayed nephropathy in the presence of novel *WT1* and *ACTN4* mutations, suggesting the potential for a new genotype‒phenotype relationship of DDS or the attenuation of disease behavior. When diagnostic testing is limited due to increased risk to the patient, we emphasize the need for personalized treatment plans and a multimodal approach with close monitoring in the long-term management of such complicated cases. This case documents novel mutations, highlights the importance of genetic testing, and justifies further investigation into the relationship between genotype and phenotype for patients with mutations contributing to renal pathology.

**Clinical trial registration:**

Not applicable.

**Supplementary Information:**

The online version contains supplementary material available at 10.1186/s12882-025-04433-4.

## Introduction

Denys-Drash syndrome (DDS) is defined by early onset nephrotic syndrome rapidly progressing to end stage renal disease (ESRD) before 4 years of age, male pseudohermaphroditism, and Wilms tumor (WT). DDS is associated with mutations in the *WT1* gene, most commonly in exons 8 or 9 [1, 2]. *WT1* mutations have also been linked to other congenital syndromes, including WAGR (WT, aniridia, genitourinary syndromes, and range of developmental delays) and Frasier syndrome, which are associated with a predisposition to WTs [2]. DDS is widely considered to be an exceedingly rare disorder, and most of the estimates of its prevalence are reserved to the number of reports in the literature. The prevalence is estimated to be < 1/10,000 and there are fewer than 500 cases ever reported; the exact prevalence of DDS is unknown [2–4]. 

The diagnosis of DDS centers around genetic testing to confirm a *WT1* mutation in the presence of associated genital abnormalities, and may include the presence of a WT at the time of diagnosis [2]. Not all children will necessarily present with a WT, and some children may present with proteinuria or steroid-resistant nephrotic syndrome (SRNS). Early identification is key in management as most children will rapidly progress to ESRD by the age of 4 [1, 3]. In some cases these children will also go on to develop a WT and require unilateral or bilateral nephrectomy. Multidisciplinary management with referrals to nephrology, oncology, endocrine, and urology are necessary to manage the renal, oncologic, and genitourinary manifestations of the disease [5]. 

This case report highlights a unique clinical presentation in a patient with DDS and genetic variants not yet reported in the literature, including a novel *ACTN4* co-mutation. *ACTN4* (α-actinin-4) mutations are implicated in various cellular processes, including cytoskeletal organization and signal transduction. These mutations could contribute to tumorigenesis by promoting cellular migration and invasion, and *ACTN4* mutations are normally associated with the development of nephrotic syndrome [6, 7]. The existence of two genetic mutations that are both associated with the development of nephrotic syndrome in this case with a unique presentation is significant. This case highlights the importance of genetic evaluation in pediatric oncology and suggests that *ACTN4* may influence the progression of renal dysfunction in patients with DDS or *WT1*-related disorders.

## Case presentation

Our patient is an 18-year-old Asian male presenting with a complex medical history that includes WT (status post right nephrectomy, chemotherapy, and radiation during infancy), DDS, chronic kidney disease (CKD) stage 2, and autism spectrum disorder that was referred to our clinic for ongoing renal management. He had recently been hospitalized for a urinary tract infection (UTI) complicated by pyelonephritis with worsening proteinuria. The patient has a history of recurrent UTIs likely related to a known diagnosis of vesicoureteral reflux grade 2.

Records indicated that the patient was diagnosed with DDS at 9 months of age by his pediatric care-team. During infancy, the patient was found to have a frame-shift *WT1* mutation with an associated WT of the right kidney; confirmed on biopsy during nephrectomy and found to have favorable histology. He also had ambiguous genitalia characterized by micropenis, perineal hypospadia, bifid scrotum, penoscrotal transposition, right-sided cryptorchidism, and persistent Müllerian duct remnants, all associated with an XY karyotype. The only feature of DDS absent in the patient at the time of diagnosis was SRNS or proteinuria. Records indicate that while the patient lacked SRNS, the patient presented with recurrent episodes of gross hematuria during infancy. While the diagnosis of WAGR syndrome (Wilms tumor, Aniridia, Genitourinary anomalies, and Range of developmental delays) could represent the symptoms seen in this patient’s presentation, the absence of defects to the iris made a diagnosis of WAGR less likely.

**Fig. 1:**
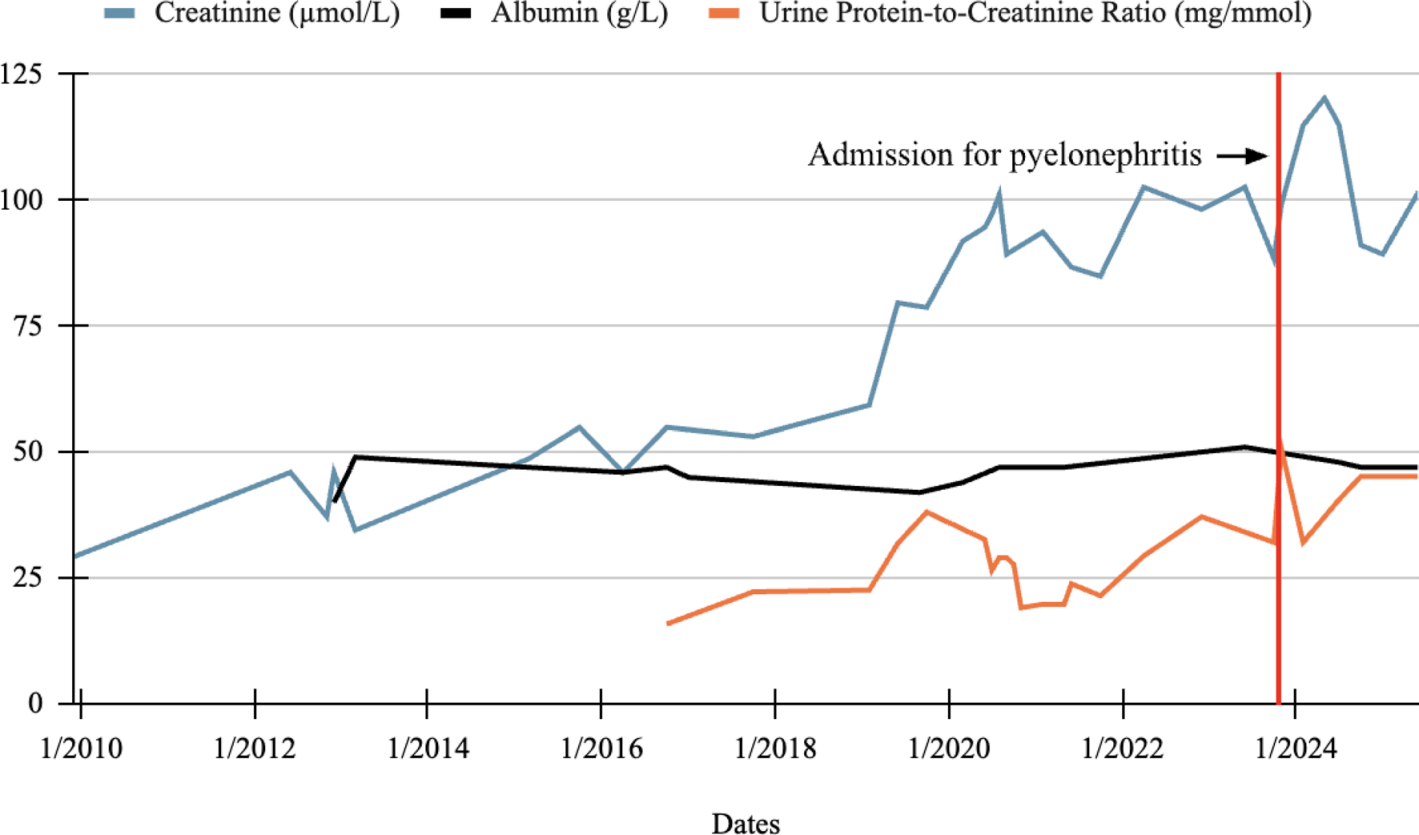
Trends in renal function over time

On presentation to our clinic, the patient was a well appearing male with a BMI of 20.06 and vital signs that were within normal limits. There were no cataracts, no papilledema on fundoscopy, and vision was grossly normal. Signs of renal-related edema were absent. At presentation, the patient’s external genitalia was normal due to surgical repair in infancy. Outside records indicated a solitary, hypertrophied left kidney, with gradually declining renal function. Note that our reporting system presents values in units commonly used in the United States, and we have provided all values in standardized international (SI) units in parenthesis. His oldest recorded baseline creatinine was 0.52 mg/dL (46.0 µmol/L), which had increased to 1.3 mg/dL (115 µmol/L) at presentation, with an estimated glomerular filtration rate (eGFR) of approximately 65 mL/min/1.73 m^2^ (1.08 mL/s/1.73 m^2^). The 2021 Chronic Kidney Disease Epidemiology Collaboration (CKD-EPI) equation was used for eGFR. His proteinuria, as indicated by the urine protein-to-creatinine ratio (URPC), also gradually worsened, rising to 0.46 mg/mg (50.9 mg/mmol) earlier that year. Figure 1  details the patient’s serum creatinine, serum albumin, and URPC over time using data from the years 2009 to 2025. An extensive review of the patient’s electronic medical record was performed, and the patient’s renal function parameters before 2009 were not available; in 2009 the patient was 3 years-of-age.

This figure shows the patient’s serum creatinine, serum albumin, and urine protein-to-creatinine ratio starting from the date range 12/2009 to 06/2025. The vertical red line at the date 10/2023 denotes the patient’s hospital admission for pyelonephritis and onset of worsening proteinuria. All available data is graphed in standardized international (SI) units. The normal reference range for creatinine is 53–110 µmol/L, albumin is 36–51 g/L, and urine protein-to-creatine ratio is less than 20 mg/mmol. Any values that were missing from the patient’s medical record were excluded. Note that exact dates are not included to ensure continued anonymity, but that all data is placed accurately within the appropriate date range.

**Fig. 2:**
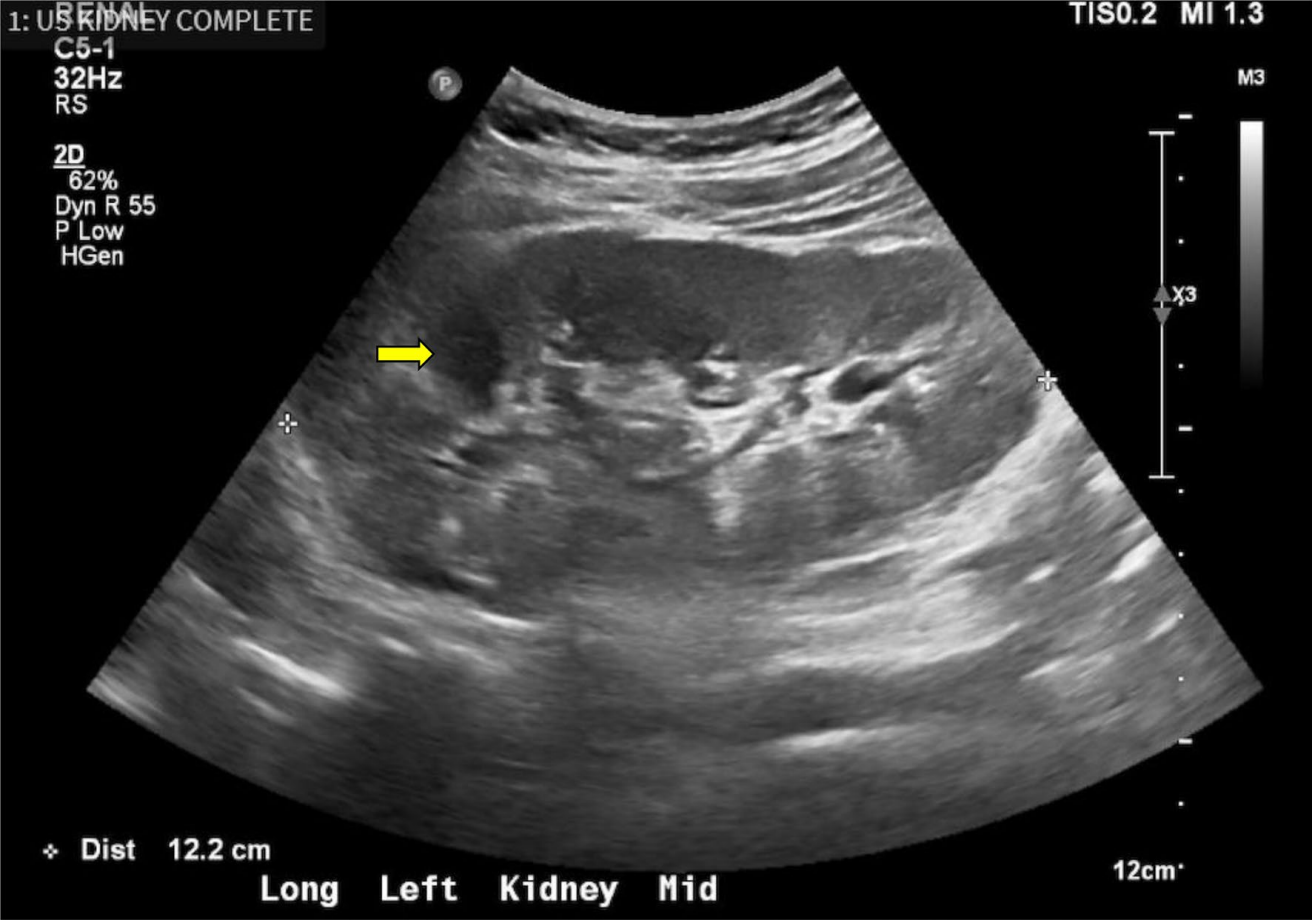
Longitudinal ultrasound image of the left kidney (coronal image)

**Fig. 3:**
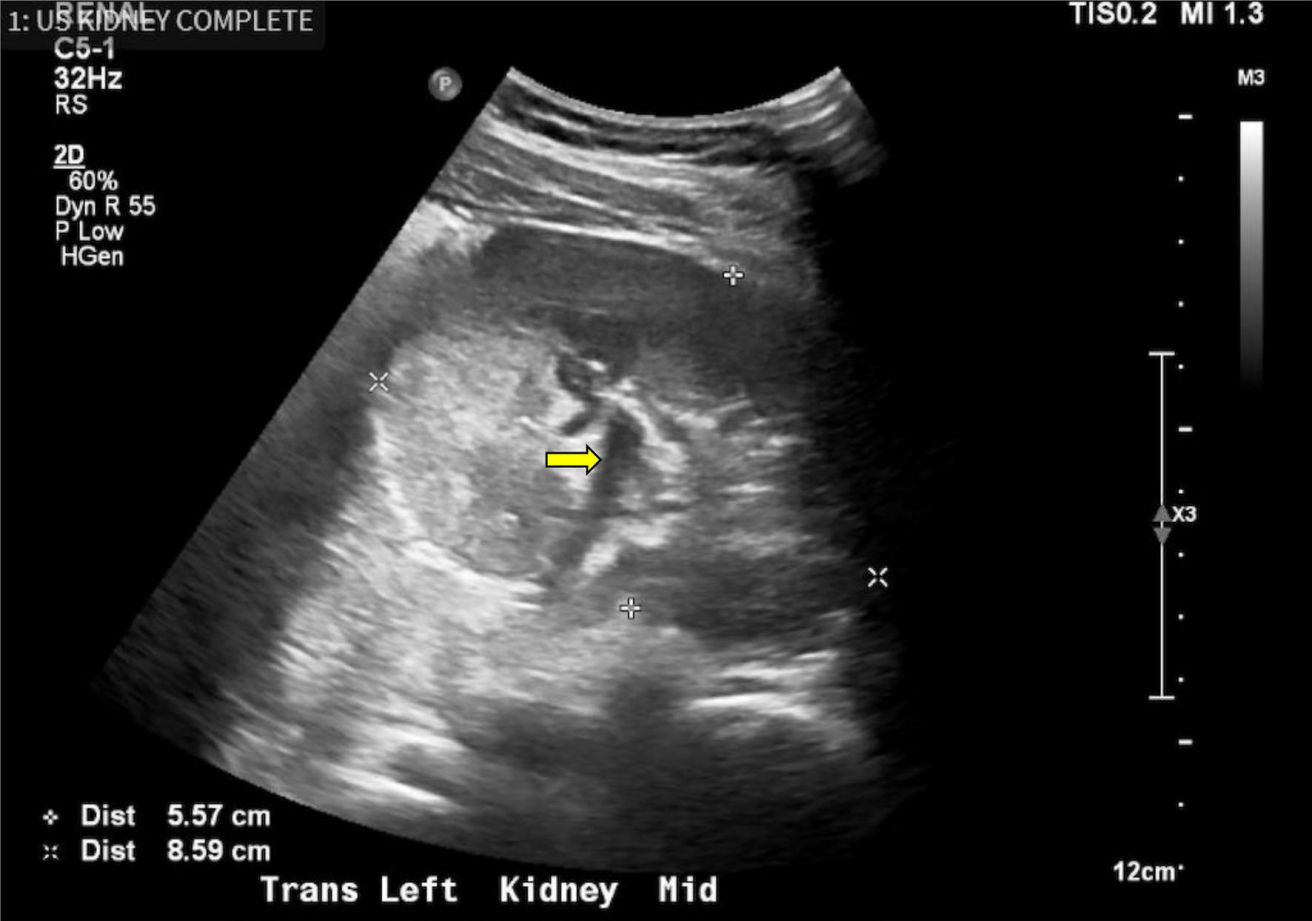
Transverse ultrasound of the left kidney (axial view)

We suspected that nephropathy was indicative of the progression of diffuse mesangial sclerosis or focal segmental glomerulosclerosis due to an underlying *WT1* mutation and DDS. The pathology report from his nephrectomy during infancy included a biopsy of the left (solitary) kidney that revealed numerous sclerosing glomeruli likely related to chronic glomerular injury. Given his solitary kidney at presentation, CKD, and previous pathology report indicating glomerular disease, we chose not to pursue a renal biopsy because of the high risk involved and minimal degree of proteinuria. Instead, we performed genetic analysis, regular laboratory tests (serum albumin, blood urea nitrogen (BUN), eGFR, electrolytes, lipid profile, URPC, urinalysis, complete blood count (CBC), coagulation profile) and renal ultrasound (Figs. 2  and 3).

The image above shows a longitudinal (coronal) view of the left solitary kidney of the patient, obtained after presentation to our clinic with ongoing proteinuria. The measurements of length can be seen in the bottom left of the image, which measured 12.2 cm. The average adult left kidney typically measures approximately 11.1 +/- 1.3 cm [8]. A benign 1 cm parapelvic cyst in the middle of the left kidney with thin septations can be observed, as denoted by the yellow arrow in the upper left side of the image.

The image above shows an axial view of the left solitary kidney of the patient, obtained after presentation to our clinic with ongoing proteinuria. The measurements of width can be seen in the bottom left of the image measured in centimeters, measuring 5.57 cm × 8.59 cm. Notably, this image also reveals some mild pelviectasis, which is indicated by the hypoechoic space within the renal pelvis, denoted by the yellow arrow in the center of this image.

Genetic analysis and laboratory surveillance constitute our best alternative for enabling early identification and intervention when worsening nephropathy is observed. Genetic analysis revealed two mutations that were likely associated with the patient’s nephropathy: a *WT1* variant, c.388_389insAC (p.Pro130Hisfs*34), and an *ACTN4* variant, c.2698T > A (p.Ser900Thr). The methodology of the genetic analysis can be found in the Supplemental Materials section. The pathology report indicated that the *WT1* mutation is likely pathogenic, and that the *ACTN4* mutation is of unknown significance. The patient’s original genetic analysis from infancy was unavailable, so it is unknown if the original report indicated the *ACTN4* co-mutation at that time. The current genetic analysis indicated that both mutations likely follow an autosomal dominant inheritance pattern, and that the patient is heterozygous for each mutation. The genetic profile of the patient’s parents was not available at the time that this case study was conducted, therefore, it is unknown if these mutations were truly inherited or occurred de-novo. However, it should be noted that the parents of the patient are 1st degree cousins, and the parents and other immediate family members are not reported to have any nephrotic syndrome or *WT1*-related disorders. The absence of *WT1*-related disorders in the family and the autosomal dominant inheritance pattern of *WT1*-related disorders is suggestive that these mutations may have occurred de-novo, but without the genotype of the parents the exact etiology is unclear.

Finally, angiotensin-converting enzyme (ACE) inhibitors were considered to address his proteinuria but were ultimately reserved until the KDIGO 2024 CKD guidelines were met: moderate-to-severe albuminuria, urine albumin-to-creatinine ratio (ACR) > 30 mg/mmol, or hypertension [9]. We considered Sodium-Glucose Co-transporter 2 (SGLT2) inhibitors for managing proteinuria, but his history of recurrent UTIs, including pyelonephritis, prompted us to hold this treatment at risk of infection. Immunosuppressive therapy, typically used in DDS for nephrotic syndrome, was also considered but ultimately avoided save for temporary use in the treatment of alternate comorbidities. In the context of this patient’s normotensive status, asymptomatic hyperuricemia, and mixed hyperlipidemia, we recommended preventative lifestyle management. The patient has been seen twice in our clinic over a year, and kidney function and hemodynamic status have remained stable under this plan; his creatinine has currently returned to 1.0 mg/dL (88.4 µmol/L). Proteinuria and the microalbuminuria ratio remain elevated without drastic fluctuations, and the patient will continue to follow up regularly at 6-month intervals with nephrology.

## Discussion

This case report aims to offer a new perspective on DDS with novel *WT1* and *ACTN4* co-mutations, providing insight into the progression of nephropathy. DDS and *WT1* mutations are typically associated with early-onset nephrotic syndrome, rapidly progressing ESRD, WT, and genitourinary abnormalities [1–3, 10, 13]. While this patient fulfilled the latter criterion, his kidney dysfunction had a much more delayed presentation. Our hypothesis is that the novel *WT1* mutation in exon 1 demonstrates a new genotype-phenotype relationship, and the presence of an *ACTN4* co-mutation may attenuate the progression of renal dysfunction that is normally observed in patients with *WT1* mutations.

Among the most common *WT1* mutation variants leading to syndromic *WT1*-related disorders, nephrotic syndrome is typically diagnosed within the first year of life, and progression to ESRD is predicted by age 3 [11–13]. To clarify, our discussion pertains to syndromic forms of *WT1*-disorders, as the early progression to ESRD in syndromic cases is not necessarily observed in non-syndromic WT [14]. In a French cohort of DDS with *WT1* variants in exon 8 and 9, it was demonstrated that 90% of the children reached kidney failure at a median age of 0.8 years and that there is a significant genotype-phenotype relationship in the presentation of DDS [13]. Our patient possesses a novel genetic *WT1* variant, c.388_389insAC (p.Pro130Hisfs*34): a frameshift of exon 1 not currently reported in the literature.

The genetic report we obtained predicts that this variant will introduce a premature stop codon at least 50 nucleotides upstream of the canonical donor splice site of exon 9 resulting in nonsense-mediated mRNA decay and complete loss of function of the protein [15]. Because nonsense-mediated mRNA decay leads to the degradation of the transcript, it would be expected that there is a complete absence of a potentially pathogenic protein. The effects caused by a complete loss of function of the *WT1* protein and its isoforms is not well represented in the literature, but likely influences the presentation of this patient’s renal dysfunction given the previously demonstrated strong genotype-phenotype relationship. The patient is heterozygous for this mutation and still possesses a wild-type allele which further complicates the clinical picture, and the patient may partially retain some degree of wild-type function. Therefore, this unique *WT1* genetic variant and the potential for residual wild-type activity more than likely contributes to atypical presentation of this patient. However, the interpretation of this patient’s phenotype is further complicated by the presence of an *ACTN4* co-mutation.

*ACTN4* mutations are associated with nephrotic syndrome with a later onset in early adulthood, with a slower progression to ESRD after diagnosis [6, 16, 17]. Additionally, *ACTN4* mutations have been previously demonstrated to have high levels of genotypic and phenotypic variability, and some individuals that carry mutations have no clinical symptoms [18]. Murine models have shown that heterozygous *ACTN4* mutations can present with mild or absent glomerular changes with variable proteinuria, and that both *ACTN4* and *WT1* mutations variably affect growth factor expression [19–21]. The interplay of these effects on growth factors may also influence the presentation of nephropathy. However, a pathogenic role cannot be attributed to the *ACTN4* mutation in this patient due to absence of supportive literature and the lack of investigation confirming the presence of the mutation in immediate relatives.

Our suspicion was that nephropathy was likely due to secondary focal segmental glomerulosclerosis (FSGS) because both *WT1* and *ACTN4* mutations are more commonly associated with FSGS than diffuse mesangial sclerosis (DMS) [2, 16, 17]. Denys-Drash generally presents with DMS, but this normally presents with nephropathy by 2–3 years of age [1–3, 10, 13, 17], which is less consistent with our patient’s presentation. Additionally, our patient’s *WT1* mutation is not a common variant, which complicates our ability to prognosticate. Regardless, we anticipate kidney disease within 20–30 years with a plan for transplant at 7% kidney function (83% currently). Thus, at this time, we focused our recommendations less on pharmacological intervention and more on lifestyle change. Given his normotensive status, referral to nutrition with recommended dietary adjustments, specifically a low-protein diet with continued monitoring of blood pressure and cholesterol, is the mainstay modality of renal preservation at this time.

Ultimately, in the context of the sparse medical literature surrounding this subject, no clear treatment guidelines are available for the longitudinal management and surveillance of this patient’s renal dysfunction. In addition, the availability of diagnostic modalities was limited, particularly biopsy, which poses a risk for thrombosis, bleeding, infections, and worsening renal function. Understanding the histologic pathology of this patient would not necessarily change management, so the risks of biopsy were too great with minimal perceived benefit. For surveillance, we are continuing to measure eGFR through serum creatinine levels, degree of proteinuria through urine protein-to-creatine ratio, urinalysis for the early detection of recurrent infection, and routine 6-month ultrasounds to monitor for cancer recurrence.

This case report is subject to several limitations. The diagnosis and management of this patient’s condition are unique to this case, thus resulting in poor generalizability to other cases of *WT1*-related disorder or DDS. Given that DDS has a significantly low prevalence, any individual presentation may not necessarily be atypical but may instead reflect ongoing efforts to further characterize the full clinical spectrum of the disease. Additionally, this patient’s *WT1* frameshift mutation (c.388_389insAC) has not been reported in the literature, and the exact significance of this mutation is unknown. While the combination of *WT1* and *ACTN4* mutations in this case of DDS may result in some degree of attenuation of disease severity, the impact of these individual, specific mutations on disease severity is also lacking in the literature. Therefore, it is challenging to predict the exact impact of these two mutations on renal function and how this patient’s disease ultimately progresses. The decision to avoid performing a renal biopsy also poses a significant limitation, as it prevents the ability to histologically examine this patient’s renal pathology. A key component in the diagnosis of DDS is histologic data, and without the biopsy, it is possible that this patient’s presentation is consistent with alternative diagnoses. Withholding SGLT2 inhibitors was based on this patient’s history of recurrent urinary tract infections and would not necessarily be applicable in other cases. Withholding ACE inhibitors in this patient does not necessarily reflect consensus guidelines and would not be recommended in other cases. Such precautions may ultimately affect our ability to medically optimize this patient and subsequently optimize his long-term outcomes. Finally, the combined effects of both mutations likely influence the long-term efficacy of our treatment plan and the patient’s clinical outcome.

## Conclusion

In this case, we present a patient with an atypical presentation of DDS characterized by delayed nephropathy in the presence of novel *WT1* and *ACTN4* mutations, suggesting the potential for a new genotype-phenotype relationship or the attenuation of disease behavior. When diagnostic testing is limited due to increased risk to the patient, we emphasize the need for personalized treatment plans and a multimodal approach with close monitoring in the long-term management of such complicated cases. This case documents novel mutations, highlights the importance of genetic testing, and justifies further investigation into the relationship between genotype and phenotype for patients with mutations contributing to renal pathology.

## Electronic supplementary material

Below is the link to the electronic supplementary material.

Supplementary Material 1

## Data Availability

No datasets were generated or analysed during the current study.

## Abbreviations

WT: Wilms tumor
ACE: Angiotensin-converting enzyme
