# Supplementary material for: Novel WT1 and ACTN4 co-mutations in a patient with Denys-Drash syndrome and an atypical, potentially attenuated presentation of nephropathy: a case report
Source: BMC Nephrol. 2025 Sep 1;26:508. doi: 10.1186/s12882-025-04433-4 (PMC12400672; doi:10.1186/s12882-025-04433-4)
Supplement: Supplementary file 1 — Supplementary Material 1 [file 12882_2025_4433_MOESM1_ESM.docx]

**Genetic Analysis Methodology**

A Renasight kidney gene panel was performed by Natera and genetic methodology was reported to the authors of this study as follows: Genomic DNA is extracted from the submitted specimen, processed and sequenced using capture-based Next generation sequencing (NGS) (minimum 20X coverage, 150bp paired-end). Sequencing reads are aligned to the human genome reference assembly, GRCh37/hg19 using an in-house developed secondary analysis pipeline, Germline Pipeline v2019.2. All small insertion and deletion variants as well as some single nucleotide variants that do not meet an internally validated quality threshold are confirmed by bidirectional Sanger sequencing. If necessary, long-range PCR followed by Sanger sequencing may be used for confirmatory testing. Copy number variation (CNV), detected by NGS, are confirmed by quantitative PCR (qPCR) or multiplex ligation-dependent probe amplification (MLPA) and analyzed relative to the normal expected copy number. Variants are annotated following Human Genome Variation Society (HGVS, www.hgvs.org) guidelines. Variants are interpreted in accordance with professional (e.g. ACMG/AMP) guidelines using literature review and other genetic resources (available upon request). Variants of uncertain significance are reported upon request while benign and likely benign variants are not reported. Variants in the PKD1 gene are classified according to the ACMG/AMP guidelines for variant interpretation. Additionally, these variants are categorized into truncating and nontruncating variants according to Heyer CM et al., (2016) (PMID:26823553). Truncating variants are defined as those that are predicted to result in a shortened, non-functional gene product. Truncating variants include frameshifting deletions, duplications, or insertions; nonsense variants; canonical splicing variants; large rearrangements (deletions, duplications, or insertions) involving at least one exon; in frame-deletions that are equal to or larger than 5 amino acids. Other single nucleotide variants that result in missense changes or in-frame deletions less than 5 amino acids are considered nontruncating.15
